# A realist evaluation of the management of a well- performing regional hospital in Ghana

**DOI:** 10.1186/1472-6963-10-24

**Published:** 2010-01-25

**Authors:** Bruno Marchal, McDamien Dedzo, Guy Kegels

**Affiliations:** 1 Department of Public Health, Institute of Tropical Medicine-Antwerp, Nationalestraat 155, B-2000 Antwerp, Belgium; 2 Volta Regional Health Directorate, PO Box HP 72, Ho, Volta Region, Ghana; 3 Department of Public Health, Institute of Tropical Medicine-Antwerp, Nationalestraat 155, B-2000 Antwerp, Belgium

## Abstract

**Background:**

Realist evaluation offers an interesting approach to evaluation of interventions in complex settings, but has been little applied in health care. We report on a realist case study of a well performing hospital in Ghana and show how such a realist evaluation design can help to overcome the limited external validity of a traditional case study.

**Methods:**

We developed a realist evaluation framework for hypothesis formulation, data collection, data analysis and synthesis of the findings. Focusing on the role of human resource management in hospital performance, we formulated our hypothesis around the high commitment management concept. Mixed methods were used in data collection, including individual and group interviews, observations and document reviews.

**Results:**

We found that the human resource management approach (the actual intervention) included induction of new staff, training and personal development, good communication and information sharing, and decentralised decision-making. We identified 3 additional practices: ensuring optimal physical working conditions, access to top managers and managers' involvement on the work floor. Teamwork, recognition and trust emerged as key elements of the organisational climate. Interviewees reported high levels of organisational commitment. The analysis unearthed perceived organisational support and reciprocity as underlying mechanisms that link the management practices with commitment.

Methodologically, we found that realist evaluation can be fruitfully used to develop detailed case studies that analyse how management interventions work and in which conditions. Analysing the links between intervention, mechanism and outcome increases the explaining power, while identification of essential context elements improves the usefulness of the findings for decision-makers in other settings (external validity). We also identified a number of practical difficulties and priorities for further methodological development.

**Conclusion:**

This case suggests that a well-balanced HRM bundle can stimulate organisational commitment of health workers. Such practices can be implemented even with narrow decision spaces. Realist evaluation provides an appropriate approach to increase the usefulness of case studies to managers and policymakers.

## Background

In the wake of the numerous global health initiatives, the health workforce of low and middle-income countries is once again receiving a lot of attention [1-3]. While the key role of health workers in improving health care quality and implementing disease control programmes is widely recognised [4,5] operational aspects of health workforce management at service provision level remain poorly studied. Indeed, the focus has been mostly on macro-level aspects, such as brain drain, the impact of human resource deficits on global health initiatives and planning and training capacity issues.

This relative neglect of studies of health workforce management explains why the current evidence base on the effectiveness of HRM policies and strategies is rather weak. More specifically, there are a number of weaknesses that limit their potential to inform decisions of policymakers or health service managers. First, the determinants of health worker performance in poor resource settings have not been studied well. Second, the HRM policies and strategies, too, are under-researched [6]. Third, systematic reviews indicate that most of the studies are methodologically flawed. A recent realist synthesis of the effect of human resource management interventions on health worker performance in LMIC found that very few studies provide adequate information on the assumptions, the context and the underlying mechanisms of these interventions [7]. The same applies to a review of the effect of HRM policies on supply, distribution, efficient use and performance of health workers [8]. Rowe and collegues came to similar conclusions [9]. Fourth, few of these studies have been carried out in LMIC [8].

All this notwithstanding, policies and management strategies are still imported from other settings into health services of LMIC without a blink of the eye. The surge of performance based financing (PBF) provides a good example. PBF is being introduced at different levels of the health system [10-13] and in a wide variety of countries, including Nicaragua [13], Cambodia [14], Rwanda [15,16], Zambia [17], Sri Lanka, Ghana, Zimbabwe, Thailand and India [18]. The evidence base, however, is very narrow [19]. Most PBF studies were found to lack controls and to neglect the analysis of confounding factors [6], which reduces the validity of the attribution of the reported effects to the intervention. Furthermore, very few studies offer indications of the conditions in which these approaches are working (see [20] for an example of a study that does).

In part, the methodological weakness of the health workforce management research resides in insufficiently rigorous studies. Some problems also stem from the widespread use of the case study. Indeed, although organisational studies is a domain marked by a lack of consensus on ontology and epistemology [21] and the consequent lack of consensus on methodology, the case study is a common research design for a number of reasons. First, it allows exploring a "phenomenon within its real-life context, especially when the boundaries between phenomenon and context are not clearly evident" [22], and thus suits well the open systems-nature of human organisations. Second, it enables investigation of organisational behaviour as it happens in its natural setting [23]. Case studies are also useful in dynamic and complex situations where multiple, interacting variables may act upon intervention and outcome [24,25]. It is well suited to research on HRM [26]. Finally, Hartley argues that case studies can help in probing and developing theory [27].

Since the publication of *Experimental and quasi-experimental designs for research *by Campbell & Stanley [28], the major limitation held against the case study design is its limited external validity, or the weak potential to generalise findings from one case to another. Other authors raise its limited attribution power: case studies are good at analysing the intervening processes or documenting evolution in time, but weak at demonstrating the causal links between intervention and outcome [29]. Much of this critique has its origins in quantitative criteria of validity, according to which case studies are based on too small numbers of cases and on non-randomised case selection, thus leading to problems of representation and inference [25]. It is exactly here that its adherents claim that theory-based methodologies can make a difference.

During the 1980s, Chen and Rossi developed the theory-based evaluation approach as an answer to policy and programme evaluation approaches that remained limited to before-after and input-output designs or that focused narrowly on methodological issues (method-driven evaluation) [30,31]. The theories of change approach [32] and realist evaluation (RE) [33] are among the most recent applications of theory-based evaluation. As we will discuss in detail below, both approaches aim at opening the black box between intervention and outcome.

For organisational research, realist evaluation seems to offer a number of advantages. It promises, first, to increase the external validity of case studies. Building upon existing knowledge, RE analyses why change occurs, or why not, and in which conditions. It aims at providing information that allows decision-makers to judge whether the lessons learnt could be applied elsewhere [34]. Repeated case studies lead to more refined middle range theories that offer increasingly refined information of context conditions, thereby increasing generalisability of such case studies [21,27,29] and improving our understanding of causal processes [35]. Second, based on its generative perspective on causality, it seeks to explain change by referring to the actors who change a situation under influence of particular external events (such as an intervention) and under specific conditions [33]. Accepting the role of actors in change (agency), realist evaluation also considers structural and institutional features to exist independently of the actors and researchers. If human action is embedded within a wider range of social processes and structures, then causal mechanisms reside in social relations and context as much as in individuals. As a consequence of this ontological perspective, evaluators need to unearth the social layers in order to understand the root causes of the problem at hand [36] and to find the mechanism that explains the outcomes of the intervention [33]. In short, Pawson & Tilley argue that realist evaluation indicates 'what works in which conditions for whom', rather than merely answering the question 'does it work?'. Realist evaluation is thus well suited to assessment of interventions in complex situations, which most organisational research is all about.

While the merits of theory-driven and realist evaluation have been amply discussed in journals on evaluation (see for instance [36-41] and [42-44], there is little documented experience in the domain of health service organisation and public health, notable exceptions being [45] and [46]. This scarcity of realist studies could be interpreted as a sign of the limited academic credibility of theory-driven evaluation in general: 'objectivist' arguments overrule 'subjectivist' research [47]. Other reasons may be practical in nature: carrying out a full-blown theory-driven evaluation is resource- and time intensive [48]. The need of assessing the underlying theory in addition to the efficacy/outcome evaluation adds to the burden [41].

In this paper, we examine whether and how a realist evaluation design can be applied in research of well performing hospitals. We present the case of Central Regional Hospital (CRH) in Cape Coast, Ghana and discuss how we applied this method, from the stage of hypothesis formulation to the synthesis of the results. This case study is part of a longitudinal study on the links between management and performance in well-performing hospitals. We describe the latter as hospitals that ensure equitable access to high quality care and that provide such services in an efficient manner. We choose CRH both because it won the award for the best hospital of the Ghana Health Service in 2004 and on the basis of previous research.

The objective of the study was to analyse the management approach at CRH. We formulated the following research questions: (1) What is the management team's vision on its role?; (2) Which management practices are being carried out?; (3) What is the organisational climate? (defined by Takeuchi et al. as the perceptions of employees regarding how the management approach is practiced and implemented in their organisation [49]; (4) What are the results?; (5) What are the underlying mechanisms explaining the effect of the management practices?

## Methods

### Principles of realist evaluation

Drawing inspiration from [34,50,51], we structured our study in 4 steps: the formulation of the Middle Range Theory, the design of the study, the data analysis and synthesis, and presentation of the results. We briefly introduce these steps from a theoretical point of view, and then describe how we developed each step in practice.

A realist evaluation research starts from a middle range theory (MRT), which is understood as "*theor [y] that lie [s] between the minor but necessary working hypotheses (...) and the all-inclusive systematic efforts to develop a unified theory that will explain all the observed uniformities of social behavior, social organization and social change" *[52] p. 39). In essence, this MRT states how the intervention leads to which effect in which conditions. Lipsey & Pollard identify different mechanisms to develop this MRT [53]. It can be formulated on the basis of existing theory and past experience. If the latter is not available, exploratory on-site research can be done to unearth the models used implicitly by the actors to make sense of the intervention - what Pawson & Tilley call 'folk theories' [33]. Through individual interviews or group discussions, the key elements of the problem or intervention, the expected outcomes and potential moderating factors are to be identified [50], p. 196). Additional information may be derived from programme or policy documents. Cause mapping or concept mapping can be used in this process [54]. Ideally, the resulting MRT is then compared with existing knowledge. A literature review identifies studies reporting other causal chains, moderating factors or unintended outcomes, allowing a plausibility check of the preliminary MRT. The result is then again discussed with the stakeholders and results in the middle range theory that will be tested. Byng constructed the middle range theory on the basis of a literature review, a description of the intervention and discussions with facilitators involved in the programmes in question [40].

Regarding designs and research methods, realist evaluation is neutral [33]: the hypothesis as expressed by the MRT is guiding the choice of data that should be collected and the methods and tools to do so. Most theory-driven evaluations in healthcare used the case study design and combine both quantitative and qualitative methods.

Pawson & Tilley call the working hypotheses that emerge during the analysis phase 'Context-Mechanism-Outcome configurations' (CMOC) [33]. Realist evaluators describe not only the intervention and its outcome, but also the context and the underlying mechanism. They seek to establish patterns or regularities that explain outcomes of interventions. In practice, the data from interview transcripts, document analysis and observation are coded with codes drawn from the initial MRT (See [40] for a practical example). Similar to other analysis methods, subsequent rounds of analysis lead to a refined set of themes, categories and codes. The emerging findings are compiled as conjectural CMOCs, which indicate how the intervention led to particular outcomes in which context and by which mechanism. Their fit with the data is checked to ensure internal validity. The retained CMOCs are then compared with the MRT, which in turn is modified if necessary [55]. In some studies, the resulting 'new' MRT was discussed with key actors in order to validate it. A new study then further refines the MRT and this cyclical process leads to accumulation of better insights in how particular interventions work, in which conditions and how [33,34].

In order to be useful in decision-making, the synthesis should present the combinations of attributes required for an intervention to be effective, a presentation of the various alternative explanations, an indication of the potential of transferability by showing the links with existing knowledge, and an indication of the preliminary nature of the findings [56].

### Formulation of our MRT

We formulated our preliminary MRT on the basis of an explorative study at CRH. During that study, interviewees indicated the importance of trust between health workers and their management, and the high levels of commitment of staff to the hospital. We also found arguments that pointed to the importance of a contingency approach to management of health workers: effective managers implement management practices that have a good fit with the nature of their workforce, the tasks of the organisation and its environment.

A second source of inspiration was our literature review of human resource management and hospital performance, which led us to high commitment management (HICOM). We retained this concept because its comprehensive approach to management fitted well with our initial analysis. The central attribute of HICOM is the combination of several complementary practices (e.g. good selection of staff, providing training on a needs basis and individual mentoring) in what is called 'bundles'. Through their research in the industrial, commercial and service sectors, Pfeffer & Veiga identified a bundle of 7 elements, which they claim is universally valid [57]: providing employment security, ensuring comparatively high compensation contingent on organisational performance, instituting training and development, putting in place selective hiring, instituting self-managed teams and decentralisation, reduction of status differences and information sharing. Organisational commitment was identified as an outcome of such HRM practices [58] and has been shown to contribute to higher organisational performance. Such balanced bundles of management practices lead to better organisational performance [59-61]. We described elsewhere the key elements of high commitment management in health care organisations [62]. Some of the mechanisms that link HICOM to better performance include positive psychological links between managers and staff, organizational commitment and trust.

We drew another element from the work of Cameron & Quinn on organisational culture [63], which points to the importance of the coherence between the vision of the managers on their role, the practices they choose to implement, and the perception of their employees of these practices. Good fit between these would contribute to better organisational performance.

A final element is the notion of 'decision space'. This concept was developed by Bossert [64] to describe the margins of freedom of health service managers at the operational level. His framework analyses how decentralisation policies affect the management practice at operational level. We retained adequate decision spaces as a potentially important context factor and a potential condition for HICOM to be possible."

It should be noted that there is considerable debate about the outcomes of HRM, and even more about the methods to demonstrate these. In general, we would describe the proximal outcomes of human resource management in terms of three categories: improved staff availability, improved staff attitudes and affects (commitment, job satisfaction) and better staff behaviour (in terms of higher task performance and organisational citizenship behaviour, and lower absenteeism). We selected organisational commitment and trust as proximal outcomes of human resource management, because our literature review pointed out that these outputs are often found to explain the effect of HICOM.

Combining all these elements with the findings of our first exploration visit, we formulated the MRT as follows:

"Hospital managers of well-performing hospitals deploy organisational structures that allow decentralisation and self-managed teams and stimulate delegation of decision-making, good flows of information and transparency. Their HRM bundles combine employment security, adequate compensation and training. This results in strong organisational commitment and trust. Conditions include competent leaders with an explicit vision, relatively large decision-making spaces and adequate resources."

### Study design and data collection tools

As will be clear at this point, we used the case study design as the basis. We collected both qualitative and quantitative data through document review of GHS and hospital records and reports, focusing on hospital HRM policies, and staffing levels and skill mix data.

In-depth interviews with all 6 members of the hospital management team (HMT) explored their management vision and practices. We based the HRM part of the interview guide on the 7 elements set of Pfeffer & Veiga [57](see some questions in Additional file 1). It must be noted that the interview guides only served as a guide to structure the interview when necessary, not as a questionnaire list that must be applied similarly in all interviews. In line with the concern that most studies focus on managers and ignore the perceptions of employees [65], we also explored the perceptions of staff regarding the management approach (the organisational climate).

In order to cover a wide range of views of different cadres, we made a purposive selection of staff. We identified the main cadres and within these, we randomly selected candidates for the interviews. This resulted in individual in-depth interviews of 3 nurses, 1 midwife, 1 doctor, 1 radiographer, 1 physiotherapist, 2 laboratory technologists, 1 clerical officer and 1 ward assistant. We also carried out 3 group discussions with heads of units, nurses, and paramedical staff (orderlies, clerical officers and account staff). Opportunistic non-participant observations were made of management meetings, ward procedures and OPD clinics.

We also developed a data collection form that focused on numbers of different cadres of staff (stocks) and on movement of personnel in or out of the hospital (flows in terms of transfer in/out, deceased staff, dismissed staff, absconded staff, retired staff).

During the preparation phase, a self-assessment of ethical issues, based on the working paper "Notes regarding ethical guidelines for health services research", of the Department of Public Health, Institute of Tropical Medicine was done. This covered the following issues: Minimal risk to participants; Invitation, information and informed consent; Feedback to interviewees and staff. We sought and obtained a written informed consent from all interviewees. Measures were taken to safeguard confidentiality and anonymity. All interviews were recorded and transcribed verbatim.

### Data analysis

We used NVivo 2.0 software for data management and analysis. The initial coding was based on a preliminary list of codes inspired by the MRT and on additional ideas that emerged during the fieldwork.

In a second round of analysis, some themes and patterns emerged (see below). In order to structure these as CMO configurations, we found it useful to borrow categories from theory-driven evaluation [66]. We described the *intervention *(in this case the HRM practices) in terms of content and application, and the intended and actual outcomes. We drew on our interviews and observations to differentiate (proclaimed) vision (what the team wants), the discourse (what they say) and the actual practices (what they do). We described the organisational climate, defined as "the atmosphere that employees perceive is created in their organisation by practices, procedures and rewards" [67]. In order to indicate how the intervention works, we analysed both the *context *and the *intervening mechanisms*, and attempted to identify the essential *conditions*.

To assess the intensity of the implementation of the practices, we developed an analytical framework based on the paper by Richardson & Thompson [59]. These authors questioned the research tools used in HRM surveys, which in their opinion often lack assessment of the intensity of application and coverage of the HRM practices. We selected coverage, intensity, internal fit and external fit as dimensions. 'Coverage' is understood as the degree to which the elements of the HRM bundle are applied to all cadres. 'Intensity' looks at the intensity of application. 'Internal fit' examines the synergistic and/or counterbalancing effect of the different elements. 'External fit' examines the appropriateness of the bundle for the cadre and organisation in question.

### Reporting of findings

The preliminary results were discussed with the management team of the hospital, and the final analysis subsequently refined. A research report was sent to the commissioner of the study, a policy brief posted on the web and the findings were presented at the 2008 Geneva Health Forum.

## Results

In this section, we present both primary findings and results from the analysis of the qualitative data in terms of the management vision, the actual management practices and the organisational climate. These sections correspond with the research questions presented above and are drawn from a 'thick' description of the case, or a detailed account of what the interviewees said, what we observed and what we learned from our document review.

### The management vision

A first element we analysed was the views of the management team members of their own role in the hospital and on how they should manage the personnel. During the interviews, the management team members did not use words like 'bundle' or 'high commitment management', but they nonetheless expressed a clear view of the hospital's roles and of how the health workers should be managed accordingly. Key terms include striving for excellence, offering services to all, attention for their personnel and sound financial management. This vision is transmitted through what they say during staff meetings or write in the mission statement and the annual reports.

This vision is well shared: not only do the director, the financial manager, the nursing manager and the non-medical administrator maintain the same discourse, also interviewees from the operational staff expressed this vision clearly, from nurses to cleaners.

*"Their vision is that, they want this place to be a first class hospital. Their aim is to save life, so that is their main focus. And whatever they want to do so that life is saved, to me is their agenda." (Non-medical worker, group discussion Non-medical staff*)

### The actual human resource management - what the management does

Based on the analysis of our interviews, observations and collected documents, we found that the actual set of practices at CRH includes more and different elements than Pfeffer and Veiga [57] listed. These authors list of seven elements includes:

- putting in place selective hiring

- providing employment security

- ensuring comparatively high compensation contingent on organisational performance

- instituting training and development

- deploying self-managed teams and decentralisation

- reduction of status differences

- information sharing

We found that selective hiring took place at the start-up of the new hospital in 1998, when the medical and para-medical staffs were almost handpicked from the pool of health workers in the region. At the time of the study (2005), however, the Ghana Health Service (GHS) regulations allowed only local recruitment of labourers and administrative staff.

The *employment security offered by the GHS *to its appointed staff was an often-mentioned reason why interviewees prefer employment in the GHS rather the private sector.

At the time of the study, setting *compensation *levels was not within the decision space of the HMT. Only financial incentives for night duties and expatriate doctors could be given. Remuneration was not linked to actual performance. Just prior to the study, health sector strikes led to the Additional Duty Hours Allowance (ADHA) policy, which significantly improved the purchasing power of the health workers - the ADHA initially constituted a mark-up of 100-250% to the salary of a doctor and of lesser proportions for other health workers.

*Training and personal development *was found to be an important part of the HRM package. A full-time in-service training coordinator was appointed and a budget allocated to organise continued medical education activities, including clinical meetings, mortality meetings, seminars and conferences. Staffs were actively stimulated to follow external courses, even during working hours and personnel from all cadres actually did.

We found *decentralised decision-making *to be a central feature. The different units enjoyed a moderate level of autonomy in terms of decision-making and objective setting. Considerable decision-making authority over a number of domains, including the highly sensitive distribution of ADHA funds, was delegated to committees composed of different cadres of staff. The management team members argued that such decision-making structure would foster active participation of staff in decisions that affect the hospital.

In this decentralised decision-making structure, we found that *teamwork *is understood as *'working all together, all engaged, all involved'*. In the daily practice of curing and caring, teamwork was most visible at operational unit level. Deliberate efforts were made to include cleaners, sweepers and auxiliary staff in decision-making.

The nursing cadre decided to introduce an all-white uniform instead of the colour-coded uniforms. Interviewed nurses indicated this *reduction of status differences *as an important policy and perceived it as a sign of respect by management. In contrast, reduction of status differences between the management team and the operational staff seemed not a concern, neither for management, nor for the staff.

*Information sharing *was one of the most striking features. Formal communication channels were in place at all levels, including regular unit and ward meetings, heads of unit meetings and top management meetings. These were complemented by the committees mentioned above. General quarterly meetings (staff "durbars"), open to all staff, offered a voice even to the hierarchically lowest cadre. Observation showed that durbars effectively contributed to low-threshold, two-way communication.

#### Additional practices

We also found that the HMT developed HRM practices not included in Pfeffer & Veiga's set: they made substantial efforts to ensure good physical working conditions, ensured good accessibility of the top managers and stressed hands-on involvement of managers and staff socialisation.

Major attention was given to *creating optimal working conditions*. The interviewees pointed to the good communication system in the hospital, the promptness of repairs, the general cleanliness of building and compound, the availability of air conditioning in virtually all rooms and the good amenities for patients. Other elements of the physical environment that were appreciated include the subsidised staff canteen, the internet café, the staff bus and the staff library. This points to the leverage of improving the working conditions. In Ghana, this may be a management intervention that increases not only the effectiveness of health workers, but also their job satisfaction.

*Top managers are accessible *for all staff. As in most Ghanaian hospitals, we found a clear hierarchy, whereby superiors should never be bypassed. Hierarchy was strong in the nursing and administrative cadres. However, interviewees mentioned the possibility to see the director or nursing manager in person when problems could not be solved with their direct supervisor. Our observations showed that staff members of any cadre effectively made use of this open door policy.

*Management stays involved at the operational level*. Interviewees reported that the nursing managers were regularly helping out staff in the wards during their twice-daily supervision rounds, while the director was still involved in clinical work. The interviews show that this was a deliberate management strategy: the top management aimed at boosting staff morale by actually working with them and by leading by example. We also found that the heads of unit steered this process by inviting senior managers and heads of other departments to their unit meetings in case of cross-border problems.

At the time of the study,*socialisation of staff *was a central element at CRH. Newcomers were given a formal induction course and rotated for a few weeks through different units before being posted to their first station. Both close supervision and peer pressure contributed to maintenance of the standards of work. Interviews show that unit heads would identify staff not following the procedures and correct such behaviour through tutoring.

#### Intensity of implementation

We analysed the actual implementation of the HRM practices with the framework we presented under Section 'Study design and data collection tools' and which was based on the paper by Richardson & Thompson [60]. First, our observations and interviews show that the elements of the HRM bundle are applied to all cadres (good *coverage*). The *intensity of application *was variable. The management team, indeed, adapts its practices in response to emerging priorities. For example, when confronted with problems of permanence of doctors at the emergency department, a custom-made incentive package was put in place. This unequal approach was not contested because all staff recognised the role of doctors in the performance of the hospital.

Second, it seems the management team reached a good *internal fit *of the bundle (good degree of synergy between elements of the bundle). There were no practices that cancelled each other out, except perhaps for the emphasis on training. This had the unintended effect of enabling staff to leave CRH for better posts. Most other elements have mutually reinforcing effects: (1) information sharing, recognition and participative decision-making; and (2) bottom-up access to management and managers getting involved in the wards.

Finally, the *external fit *of a HRM bundle is the fit of the management practices with the core activities of the hospital (caring and curing) and with the mission of the organisation (providing accessible quality care). The HRM practices stimulate good professional practice by nurses, midwives and doctors by providing adequate autonomy to the operational units regarding their daily activities, while ensuring coordination between these units. The management is also perceived to provide effective support, information and resources (see below). As such, the bundle fits well to the task and mission of the hospital and to the professional values.

### The organisational climate: the management practices as perceived by the staff

Four themes emerged in the analysis of the perceptions of the operational staff of the HMT's actions: team work, strong perceptions of support by the management team, recognition and trust. As we will discuss below, these themes point to mechanisms that help explain how the management strategies worked.

#### Teamwork stimulates staff from all cadres to be involved in care

The interviews indicated a strongly shared feeling among staff members that team work matters: they maintain that quality of care can only improve if all types of staff are involved.

*"In some places, nobody gets close to the Nurse Manager and it is like she only decides what she wants at the place. (...) But here, everybody is important. We see everybody's job as important aspect of the health care delivery system, so we include everybody in the care." (IO 1, Unit head, Ind. interview*)

Junior staff members pointed out the 'free' relations with their superiors.

*"We are all free in our units. My head always comes round to see what is going on over here. If something is not in the right place, he will show you to do this or that. So, always the heads are helping us, so we also feel free to work with them." (Non-medical staff, GD Non-medical staff*)

*'Free relations*' strengthen the collaboration between operational staff and their heads of units, but also with the top managers. Interviewees similarly mentioned the easy communication between the middle line staff and the HMT.

*"I would say there is good relationship both formally and informally. We communicate by memos, but as soon as I came, I can just walk straight to Director and tell him: 'This is the problem', and we just brainstorm to see how the problem can be solved." (IM5, HMT member, Ind. interview*)

#### Perceptions of support by the management team

Interviewed staff members often mentioned that they feel supported by the HMT. First, interviewees expressed the feeling that the HMT is effectively solving problems. Unit meetings or ward conferences are a good example of how formal meetings can prevent or solve coordination problems.

*"The ward conference is very good. The accountant is there, the pharmacist is there, the lab man is there, everybody is there. The meetings or presentations are not for fault finding. We pick issues from there and we make our corrections or cover loopholes." (Head of unit 1, GD Unit heads*)

Informal and non-structured opportunities exist, too, and are used to good effect. Interviewees pointed out how open relationships and good access to top managers allows them to take a problem to the 'next level'.

*"As a unit head, if I think that something is not going on well, my demands are not being met, l can approach the director and we sit down and talk about it. (...) You are free to enter his office anytime to discuss your problem, especially when you think things are not going on well" (Head of unit 4, GD Unit heads*)

Staff members appreciated not only the possibility to discuss work-related problems with their superiors, but also the attention given by the latter to their professional development. This also applies to members of the hospital management team.

*"He [the director] made every opportunity for my career advancement. He is always looking out, listening and trying to help where he can, to see how he can help people to progress. So, when you have someone doing that for you, at least you also have to return the same to him." (IM5, hospital management team member, Ind. interview*)

#### Strong perception of recognition

The interviewees expressed strong feelings of recognition by the management team. They explained how a range of practices, from a word of appreciation to tangible rewards expresses the appreciation of the HMT for their work.

*"At the end of the year, every staff here is given a token. Sometimes, something in the form of food, money, a get-together, occasionally words of motivation, a tap on your shoulder, meeting you and finding out how is it, how is the work going on. This serves as motivation." (IO7, Head of unit, Ind. interview*)

Interestingly, several interviewees mentioned the initial staff selection, when the hospital was started up, as a key event, not only because it helped set standards, but also because of its strong undertone of recognition.

*"To start with, I can surely say that, the standard that was set right from the inception of the hospital has made such a mark. Because immediately when this hospital was instituted, we were to come for an interview. So, a high standard was set (...) and they see if you have the call to work. On that note, in coming out to publish the names of those to come here, it is like Government releasing a white paper. By that time, you feel as if you are in heaven. (...) With that alone, that standard was set and everybody was expected to give of his best." (Nurse, GD Nurses*)

#### Perceptions of trust

We explored the issue of trust, which we found to be an important element in the explorative study, by asking staff how they would rate the levels of trust at CRH and how they believe trust is generated. The interviewees indicated fair levels of trust both amongst staff and between management and staff.

*In the whole hospital, there is some trust, but I don't think it is 100%. May be it is between 80% - 95%." (Unit head 3, GD Unit heads*)

Asked how management practices influence the levels of trust, they pointed out the importance of meetings during which information is exchanged, the willingness of managers to discuss decisions and the resulting perception of transparency.

*"At least, we have management meetings and after that, management meets the unit heads and tells them what the institution wants to do, the programmes they have embarked on. They discuss with the unit heads and if somebody does not understand something, management explains it. The unit heads are supposed to go down and explain to their subordinates. And when we have staff durbars, these things are also brought up. So, transparency is there, we can understand things. Anything they want to do is explained to workers. (Unit head 3, GD Unit heads*)

These consultations and opportunities to discuss important issues contribute to perceived fairness of the decisions. Interviewees said that less rumour mongering and suspicion arise when people are informed why certain measures are implemented and others not.

*"At the end of the day, like we had our last year's meeting after we presented our reports, management too presented their report, their financial report, what they got and how they spent their expenditure and those things. So, there will be no room to think that somebody is cheating on you, or management is hiding certain things from us. So, we know what is happening, you don't need to or there is no room for suspicion. (...) I think it is a fair deal between management and staff." (IO2, Midwife, Ind. interview*)

Another source of trust is the effective support staffs receive from their superiors in case of problems.

*"Even the Director himself came here three days ago. So, what he said, he has done it. That is why I say I trust him." (Non-medical worker, GD Non-medical staff*)

*"The trust comes from the urgent action taken when there is a problem. If there is any problem on discipline for example, an ad-hoc committee is set up and within days, the matter is settled." (IM4, HMT member, Ind. interview*)

## Analysis

After categorising, and thus making sense, of the primary data in the form of CMO Configurations, a realist evaluation seeks to examine the link between these findings and the middle range theory it set out to examine. In practice, we searched for potential causal pathways between the management practices and the apparent outcomes of commitment and trust. To do so, we summarised the above findings and then searched for CMO configurations.

### A summary of the intervention and its outcomes

Our interviews and document review show that the Hospital Management Team identified good hospital performance as the intended distant *outcome *of its management practices and a motivated and well-performing workforce as the proximal outcome. As mentioned above, the scope of this study did not allow examining the association between management practice and hospital performance, and we focus on the effect of these practices on organisational commitment and trust, the proximal outcomes we retained on the basis of our preliminary theory-building.

The *actual *intervention can be summarised as a combination of HRM practices: socialisation of (new) staff, training and personal development, good communication and information sharing between different levels of the organisation, and decentralised decision making to the level of ward and department teams. We also found important additional management practices: the creation of good working conditions, the good accessibility of top managers, and the active involvement of the manager on the work floor.

Regarding the *process of implementation*, we noted a good coherence between the HRM practices and the management team's vision. Indeed, in line with their vision, the management team motivates the staff through different interventions: remuneration, effective support and recognition. The HRM practices are reinforcing each other (good internal fit). The bundle is well adapted to the different cadres of a healthcare organisation and its mission (good external fit). It is applied similarly to all cadres (good coverage). The intensity is variable, but this poses no problems for the staff.

Realist evaluation improves external validity of a case study by describing the *implementation context*. During the study, we found several potentially important elements in the context of Central Regional Hospital. First, as testified by the brain drain, Ghana has a well-trained health workforce from which the GHS (and thus CRH) can draw personnel. Its medical and paramedical cadres display a high degree of professionalism, and there is a general culture of professionalism in the GHS. Second, reasonably good resource availability in terms of hospital funding and management capacity allows investing in the workforce. Indeed, commitment eliciting management practices are costly, especially in management time and in terms of training costs.

We found that the *outcomes *of the HR management bundle at CRH included trust, commitment and strong perceptions of recognition and of support by management, which result in a positive organisational climate.

### CMO configurations

During the later phases of the analysis, we found that the management practices can be grouped according to their key mechanism and this led to the description of two parallel CMO configurations, each with their own outcome.

The first CMO can be summarised as 'keeping up standards of excellence through organisational culture'. The hospital had a head start: staff members were selected on professional and motivational grounds by the management team. This lengthy selection procedure gave the staff a feeling of belonging to an elite corps of health professionals and reinforced their professional identity. The management team used this opportunity to initiate a culture of high standards of professional excellence. They set up an induction programme for new staff, and much attention was paid to teamwork and supervision. This reflects findings of Schein [68]: such practices serve as strong embedding mechanisms of the organisational culture. There was equally much attention for a clear role distribution and for task monitoring. In summary, both 'hard' and 'soft' management practices are balanced in the bundle. The former include general rules and procedures, task distribution for clinical and administrative staff and monitoring of task performance; the latter include induction courses, peer pressure mechanisms and training/personal development opportunities. All this reinforced the initial capital of professional excellence. Availability of a pool of professional health workers is an important context element, and may be essential for such a bundle to work.

The second CMO configuration can be summarised as follows: a hospital management team can attain higher organisational commitment if it strengthens positive reciprocity relationships that are based on social exchange, even in hospitals with limited HRM decision spaces. Key practices in this set include creating open access to managers for all staff and grass-root involvement of managers at operational level. This reinforces open relationships and contributes to solving operational problems and conflict resolution. In turn, it stimulates the feeling of perceived organisational support. Eisenberger and colleagues describe this as the beliefs and perceptions of employees regarding the support provided and the commitment demonstrated by the organisation in their staff [69]. Employees interpret decisions and actions of their managers and their trustworthiness in terms of the commitment of managers to their staff. At CRH, the leadership and management style is indeed perceived to be effective (in meeting its promises and in ensuring adequate physical working conditions) and supportive, even on the personal level. Ultimately, such practices stimulate reciprocity and as a result, organisational commitment. This in turn contributes to organisational performance [70].

Availability of well-trained health workers and adequate funding seem intuitively to be *essential *context elements in both CMO configurations.

### The new MRT

Our analysis identified two CMOCs that indicate causal pathways between sets of HRM practices and HRM outcomes, and we modified the MRT accordingly:

"The management of a well-performing hospital deploys organisational structures that allow decentralisation and self-managed teams and stimulates delegation of decision-making, good flows of information and transparency.

In the management of health workers, they implement a balanced bundle of management practices that includes both clear goal setting, role distribution and task monitoring (hard HRM) and training, support and recognition (soft HRM).

Based on the mechanism of perceived organisational support and reciprocity, such combinations lead to a positive organisational climate that includes recognition, respect, commitment and trust. If these are taken up into the organisational culture and newcomers are inducted into the OC, enduring effects of such practices can be expected.

Conditions for such management practices to work include competent leaders with an explicit vision, a minimum of resources and conducive institutional arrangements, including effective decentralisation and appropriate decision spaces (although the latter can be narrow for HRM)."

## Discussion

On the basis of this one study, we cannot yet draw firm policy recommendations. Nevertheless, it offers interesting insights in health workforce management and in the use of realist evaluation.

### Lessons for policy and practice

First, we found a proof of concept for HICOM in resource-poor health services. Second, our study found variant practices compared with the bundle described by Pfeffer and Veiga, which supports the findings of Richardson & Thompson [60] and Marchington & Grugulis [71]. Third, this case reinforces the point that in management of health workers, we need to apply coherent bundles of practices, and not focus on singular interventions. In HRM, the quality of management practices counts more than the quantity. It is not the actual number of practices, but rather the process by which these practices are put in place that is related with positive staff attitudes like commitment, job satisfaction and procedural justice [65]. This is in line with conclusions of other studies in other sectors [26,72].

Regarding the mechanisms, our findings relate to the analysis of Evans & Davis [73], who situate the underlying mechanisms of high commitment management at the level of the internal social structure of the organisation. Such practices improve knowledge, skills and abilities, but they exert also major effects at the level of relationships. Weak ties are strengthened [74], reciprocity is established and maintained [75] and shared mental models contribute to a strong organisational culture. This in turn affects behaviour of staff and improves organisational efficiency and flexibility, and ultimately, organisational performance. The evidence of the impact of such reciprocity relations or of organisational commitment on organisational performance is not strong, and further research should investigate whether and how high commitment leads to better performance in healthcare organisations.

We found that the decision spaces managers require to develop a responsive HRM approach may be smaller than is often thought. At the time of study, the decision spaces of regional hospital managers in Ghana were quite limited concerning HRM. As important as the formal decision space is its actual utilisation. At CRH, the team exploited its decision spaces well to create its own way of management within the defined institutional arrangements of a 'regional hospital' (e.g. by using committees and delegation of decision-making power).

Finally, a balanced management approach is costly, especially in management time. It requires reasonable financial resources and a management capability to deal not only with administration but also with the less tangible issues of relationships, organisational culture and motivation of staff.

Future research should establish what other HRM approaches lead to high commitment, under which conditions HICOM works, and how it can be stimulated. This last question deserves attention. Health services in many LMIC are both ill equipped and not sufficiently supported to implement a HRM approach that differs from a mere administrative approach. In the first place, the managers of health services are mostly medical doctors. Human resource management is not an element of the medical education curriculum. Even if they received additional public health or management training, the curriculum mostly equates HRM to personnel administration and this hardly prepares future health service managers for responsive management.

### Methodological lessons

In this case study, we used a realist evaluation approach because we consider health care organisations to be essentially social entities. Pawson argues that realist evaluation is well suited to investigate change in such social systems [34]. Its focus on the generative causality that underlies interventions, stimulates the analysis of how the intervention works and in which context conditions. This results in more detailed conclusions that indicate how the intervention was carried out, which effect it had and how it worked. It also offers insights in the context elements. Such theory building helps to overcome the limits of traditional case studies, and specifically their low external validity and low power to explain change [42]. However, appealing as it is, realist evaluation poses a number of challenges for the researcher.

#### The attribution paradox

Perhaps the most critical issue is the attribution paradox. Because of its ontological and epistemological basis, realist evaluation is quite fit to assess complexity [76,77] and may contribute most in research of exactly such topics. However, research of complex problems needs to confront multi-causality. In complex systems, the behaviour of people and organisations alike is determined by many interlinked factors. Health professionals act under influence of their professional norms, social pressure, management interventions, and not least, their intrinsic motivation. Assessing the exact contribution of a set of management practices to overall organisational performance may therefore be virtually impossible.

What realist evaluation *can *do is to stimulate the researcher to describe a detailed picture of the causal web that includes the multiple determinants and to categorise these as intervention, underlying mechanism or essential context factor. In our case, we have arguments to say that both commitment-eliciting management and personnel administration are required, but we cannot (yet) indicate which among these two sets is the most important in which setting.

The conclusion may be that one needs to accept that the kind of evidence provided by realist evaluation can never be put in the same categories of evidence produced by controlled experimental methods, not only because of its perspective on causality, but also because of the complexity of the subjects on which it will be applied.

#### The MRT fallacy

While any researcher adopts specific reference frameworks during her research, realist evaluation asks researchers to make these frameworks explicit in the form of a MRT. This implies a risk of developing a tunnel vision: the researcher may remain blind for the unexpected factors and alternative explanations. This risk can be reduced by the plausibility check during the development of the initial MRT, triangulation of findings, analysis by multiple researchers and discussion with stakeholders and peers.

The MRT fallacy also operates at the stage of analysis and of dissemination. During analysis, we did several rounds of plausibility checks, because we kept finding alternative explanations in disciplines such as organisational psychology, organisational theory and sociology. The CMOCs and resulting MRTs are indeed most often just one way of explaining the findings. A middle-range theory can indeed never cover *all *possible explanations of change [34]. In Pawson and Tilley's view, a realist evaluator does not strive at nor pretend to provide the ultimate evidence that the intervention works. Rather, she aims at enlightening the decision-maker, a process of utilisation of research that may be the most frequent in case of social science [78]. In such cases, a pragmatic position should be taken, whereby one tries to refine the middle range theory as much as practically possible, with the explicit aim of providing options for improvement rather than reaching a perfect understanding of the intervention as such [56,79].

#### The CMO dilemma

As we mentioned, the CMO configuration is a powerful model to go beyond the classic case study, as it forces the researcher to go beyond description. However, a true application of realist evaluation requires not only a systematic description of the intervention in terms of intervention, outcome, context and mechanisms. Also the generative causal relationships between these elements need to be assessed. In our analysis, this proved difficult at several levels.

The first important hurdle is the differentiation of the effect of the context from that of the intervention. This feeds the attribution paradox: is the outcome the result of the intervention - and to which degree - or are there context elements that explain the change in outcome - and to which degree? Furthermore, some context elements can be expected to moderate the relation between intervention and outcome, and in some cases, the outcome of an intervention will influence its context (initiating feedback). Regarding our case, probably more attention needs to be given to the role of professionalism. Professional values can steer providers' behaviour to an important degree and could partially explain the behaviour of certain staff, irrespectively of the management strategies. Most likely, we may find that the observed management strategies are in a close fit with professional behaviour traditions.

Secondly, the realist researcher seeks to describe the mechanism that is triggered by the intervention and that leads to the outcome. Confusion may result from the finding that some context elements are essential for the outcome: is this context element then part of the mechanism? We clarified this issue by considering context elements as actors or factors that are external to the intervention - that are present or occurring even if the intervention does not lead to an outcome -, but which nevertheless may have an influence on the outcome. The mechanism is the causal pathway that explains how the intervention leads to an observed outcome *in a particular context*. In other words, the intervention leads to an outcome in specific contexts if it triggers certain mechanisms. If the mechanism is found to be context-dependent, which in health services may often be the case, essential context elements can be identified. In our case, the professionalism of the staff selected to work at CRH is a context element, the decision to introduce an induction training was a management decision, and the effect of building an organisational culture was a mechanism.

#### The efficiency question

By its very nature, RE may yield information that is particularly useful for policymakers. However, by its same nature, a RE needs considerable expertise and ample time and resources, because of its comprehensive scope. Indeed, besides the efficacy/outcome evaluation, also the underlying theory and the context must be accounted for [41,80]. In our case, work at CRH started in 2004 with an exploratory visit, and much analysis went on after the second visit in 2005 and the third visit in 2007. Such timelines may still be acceptable in case of non-urgent issues, but far less in case of high-interest policy issues.

## Conclusions

Realistic evaluation offers a comprehensive approach to assessment of interventions in complex situations that can go beyond the simple efficacy question. We developed a realist case study that unravelled the management practices put in place by a hospital management team in Ghana. This study shows that it is possible to implement high commitment management practices in LMIC and that these are perceived to be relevant by the health workers. We found that through a well-balanced bundle of HRM practices, management teams can stimulate organisational commitment and an organisational culture of excellence. At CRH, the HRM bundle included sound administrative management. Reciprocity and perceived organisational support emerged as an important underlying mechanism. In applying the realist methodology, we also encountered a number of pitfalls and paradoxes. Only through further practical applications will we find out how these can be overcome.

## Competing interests

The authors declare that they have no competing interests.

## Authors' contributions

All three authors contributed to the original design and analysis. BM and MD carried out the data collection. BM and MD analysed the data. BM, MD and GK contributed to the discussion section and to writing the manuscript. BM edited the final draft. All authors read and approved the final manuscript.

## Pre-publication history

The pre-publication history for this paper can be accessed here:

http://www.biomedcentral.com/1472-6963/10/24/prepub

## Supplementary Material

Additional file 1 Examples of interview questions from the interview guide Click here for file
