# Supplementary material for: A realist evaluation of the management of a well- performing regional hospital in Ghana
Source: BMC Health Serv Res. 2010 Jan 25;10:24. doi: 10.1186/1472-6963-10-24 (PMC2828434; doi:10.1186/1472-6963-10-24)
Supplement: Additional file 1 — Examples of interview questions from the interview guide [file 1472-6963-10-24-S1.DOC]

**Additional file 1 – Examples of interview questions from the interview guide**

- Your hospital was awarded the national award for the best regional hospital. Why is this hospital functioning relatively well?

- What is the hospital doing to retain its staff and what to attract new staff?

- How do staff members feel about working at the hospital?

- What attracts professionals to come to work at CRH in your opinion?

- Would you recommend this hospital to a colleague who is looking for a job? If so, Why?
